# Insight into *k13*-propeller gene polymorphism and ex vivo DHA-response profiles from Cameroonian isolates

**DOI:** 10.1186/s12936-016-1622-x

**Published:** 2016-11-26

**Authors:** Sandie Menard, Joëlle Njila Tchoufack, Christelle Ngou Maffo, Sandrine E. Nsango, Xavier Iriart, Luc Abate, Majoline Tchioffo Tsapi, Parfait H. Awono-Ambéné, Francis A. Abega Mekongo, Isabelle Morlais, Antoine Berry

**Affiliations:** 1 Centre de Physiopathologie de Toulouse Purpan, INSERM U1043, CNRS UMR5282, Université de Toulouse, Toulouse, France; 2 Laboratoire d’Entomologie Médicale, Organisation de Coordination pour la lutte contre les Endémies en Afrique Centrale, Yaoundé, Cameroon; 3 Faculté de Médecine et des Sciences Pharmaceutiques, Université de Douala, Douala, Cameroon; 4 Service de Parasitologie-Mycologie, CHU Toulouse, Toulouse, France; 5 UMR MIVEGEC, IRD 224-CNRS5290-UM, Institut de Recherche pour le développement, Montpellier, France; 6 Dispensaire de Nkol-Eton, BP894 Yaounde, Cameroon

**Keywords:** Artemisinins, Dihydroartemisinin, Resistance, *Plasmodium falciparum*, Ring-stage survival assay, K13, Clearance, Cameroon

## Abstract

**Background:**

The spread of *Plasmodium falciparum* resistance to artemisinin derivatives in Southeast Asia is a major source of concern and the emergence of resistance in Africa would have dramatic consequences, by increasing malaria mortality and morbidity. It is therefore urgent to implement regular monitoring in sentinel sites in sub-Saharan Africa using robust and easy-to-implement tools. The prevalence of *k13*-propeller mutations and the phenotypic profiles are poorly known in sub-Saharan Africa. Here, the *k13*-propeller polymorphism was compared to both ex vivo susceptibility to DHA and early parasitological and clinical responses to artemisinin combination therapy (ACT).

**Methods:**

*Plasmodium falciparum* isolates were collected in 2015 in Yaoundé (Cameroon) from patients treated with dihydroartemisinin-piperaquine combination. Samples were analysed for their susceptibility to artemisinin using the *k13*-propeller sequencing, the ex vivo ring-stage survival assay, the in vivo parasite positive rate and the clinical statute at day 2.

**Results:**

None of the collected isolates revealed the presence of resistance mutations in the *k13*-propeller sequence. The median ring-stage survival rate for all the 64 interpretable isolates after a 6-hour pulse of 700 nM dihydroartemisinin was low, 0.49% (IQR: 0–1.3). Total parasite clearance was observed for 87.5% of patients and the remaining parasitaemic isolates (12.5%) showed a high reduction of parasite load, ranging from 97.5 to 99.9%. Clinical symptoms disappeared in 92.8% of cases.

**Conclusion:**

This study demonstrated the absence of *k13*-resistant genotypes in *P. falciparum* isolates from Cameroon. Only synonymous mutations were found with a low prevalence (4.3%). A good association between *k13* genotypes and the ex vivo ring-stage survival assay or parasitological and clinical data was obtained. These results give a baseline for the long-term monitoring of artemisinin derivative efficacy in Africa.

## Background

Malaria remains a major public health problem largely due to *Plasmodium falciparum* drug resistance. Artemisinin (ART)-based combination therapy (ACT) is the first-line treatment for falciparum malaria, used worldwide to ensure the highest cure rates and to reduce the spread of drug resistance [1]. Unfortunately ART-resistant *P. falciparum* was reported on the Cambodia/Thailand border in 2008, characterized by a reduced parasite clearance in patients as primary parasitological feature [2]. ART-resistance has spread and today the resistance has been confirmed in five countries of the Greater Mekong Sub-region [3–7]. The identification of ART resistance in Asia is reminiscent of previously emerged resistance to chloroquine and sulfadoxine-pyrimethamine, which appeared in the same region and rather quickly reached Africa with dramatic consequences of increased malaria mortality and morbidity [8, 9]. The only question for Africa is to know when and where resistance to artemisinins will appear. It is, therefore, urgent to implement regular monitoring in several sentinel sites in sub-Saharan Africa using robust and easy-to-implement tools.

In the presence of ART derivatives, resistant parasites are able to stop their life cycle in ring stage and enter in quiescence [10–12]. Because of this particular mechanism of resistance, different from other anti-malarials, the standard determination of 50% inhibitory concentration (IC50) is not informative for assessing the level of resistance of *P. falciparum* isolates to ART derivatives. Today, in addition to clinical trials, two new tools are available for surveillance of ART resistance: (i) the detection of single nucleotide polymorphisms (SNPs) in the PF3D7_1343700 kelch propeller (*k13*-propeller domain) that have been identified as a key causal determinant in Southeast Asia [13, 14]; and, (ii) the ring-stage survival assay (RSA) [15] that gives phenotypic information, evaluating by microscopic counting the proportion of viable parasites after a 6-hour exposure to 700 nM dihydroartemisinin (DHA).

A total of 186 different *k13* alleles have been reported [16] in Southeast Asia so far. In Africa, different non-synonymous *k13*-propeller-region mutations have been reported but remain rare and highly diverse [17–19]. Moreover, these SNPs were different from those in Asia and have never been associated with increased parasite half-life values or clinical failures [19–22]. So the monitoring of ART susceptibility must be implemented in Africa but it will require the establishment of a baseline correlation between genotypic and phenotypic data. Furthermore, to date only one study performed in Uganda evaluated the ex vivo RSA associated with *k13* polymorphism on few isolates (n = 43), and this study did not report any increased survival rate associated with non-synonymous *k13* mutation [17].

In this work, the polymorphism in the *k13*-propeller domain of *P. falciparum* isolates from Yaoundé, Cameroon was evaluated and these genotypic data were associated to the in vivo and ex vivo phenotypic results. Thus, all these results provided baseline data of survival rate profiles according to the *k13* gene polymorphism of African *P. falciparum* isolates.

## Methods

### Study site and design

A prospective study was carried out between March and June 2015 in the Nkol-Eton healthcare centre in Yaoundé *intra*-*muros*, Cameroon. A finger-prick, Giemsa-stained, thick smear was performed on all patients with symptoms of malaria to assess the presence of *P*. *falciparum* asexual parasites. Patients ≥4 years old, with uncomplicated falciparum malaria and >1000 asexual parasites per μl on positive thick blood smear, were enrolled as volunteers after they or their parents had signed an informed consent form. Patients who had any anti-malarial treatment within the 15-day period before falciparum diagnosis were excluded from the study. Patients with severe malaria (defined by WHO criteria) or with vomiting preventing any oral treatment were referred to the hospital for adequate care and were not included in the study. At enrolment (day 0), study subjects’ parasite density, body temperature and body weight were recorded along with any other symptoms and signs.

The patients enrolled in the study were given a three-day course of DHA 40 mg/piperaquine (PPQ) 320 mg (Malacur®, Elder Pharmaceuticals LTD, India) according to recommendations. The prescription was based on body weight (10 to <20 kg: 40 mg DHA + 320 mg PPQ or one tablet; 20 to <40 kg: 80 mg DHA + 640 mg PPQ or two tablets; ≥40 kg: 120 mg DHA + 960 mg PPQ or three tablets). To ensure good treatment compliance the patients took the first dose of DHA/PPQ in Nkol-Eton healthcare centre in presence of a nurse and went home with the second dose. Twenty-four hours after enrolment, a phone call to patients ensured that the second dose was well taken. The last third dose of DHA/PPQ was given during the follow-up visit on day 2 at the Nkol-Eton healthcare centre in presence of a nurse. In case of treatment failure, a standard replacement anti-malarial therapy (quinine salts) was administered according to WHO recommendations.

### Ethics statement

All procedures involving human subjects used in this study were approved by the Cameroonian National Ethical Committee (statement n°: 2015/04/582/CE/CNERSH/SP).

### Ex-vivo ring-stage survival assay (RSA)

Venous blood samples were collected into acid-citrate-dextrose (ACD) vacutainers at day 0 before treatment, and were kept at 4 °C until processing. The ex vivo RSA was performed directly from the ACD blood sample within 24 h after blood collection, as previously described [15]. Briefly, after elimination of plasma, white cells and anticoagulant, *P. falciparum* parasites with a parasitaemia between 0.1 and 1% were exposed to either 700 nM DHA or 0.1% dimethyl sulfoxide (DMSO) (DHA solvent) for 6 hours, washed and then cultivated for 66 h at 37 °C under humid, oxygen-deficient atmosphere (candle jar). Microscopic quantification of the proportion of viable parasites was performed by expert microscopists on Giemsa-stained thin smears to calculate the growth rate as the ratio of parasitaemia between DMSO-exposed and initial conditions, and the survival rate as the ratio of parasitaemia between DHA-exposed and DMSO-exposed conditions. Survival data were considered interpretable only if the growth rate was ≥1. Equipment necessary for implementation of RSA included laminar flow hood, candle jar (without gas), −20 °C freezer, 4 °C fridge, and cold chain.

### Sequencing of *k13*-propeller domain

DNA was extracted from venous blood samples obtained at patient enrolment (day 0) using the spin protocol of QIAamp Mini kit® (Qiagen, Hilden, Germany) according to manufacturer’s recommendations. PCR amplification and sequencing of *k13*-propeller domain were performed, as previously described [13]. After control on 1.5% agarose gel electrophoresis, the PCR products were sequenced by ABI 3130xl Genetic Analyzer. Sequences were compared to 3D7 reference strain sequence with BioEdit Sequence Alignment Editor (version 7.2.3).

### Early clinical and parasitological responses to treatment

Patients were asked to return to the healthcare centre for follow-up visits on day 2 after treatment. Each visit included completion of a standardized history form, a physical/clinical examination for recording any signs (temperature) or symptoms (headache, vomiting, diarrhoea, diffuse pain), and a finger-prick blood sample for thick smear. Blood smear slides were stained with a 10% Giemsa solution and examined microscopically under oil-immersion at 1000×. Parasitaemia was determined by counting the number of asexual parasites against 500 white blood cells and the density estimated considering 8000 white blood cells per µl. Slides at day 2 were assessed independently by two expert microscopists. A third microscopist validated any case of discordance.

Clinical and parasitological responses to treatments were classified as follows: (1) parasite negativity rates (PNRs) for the proportion of patients without any parasitaemia on day 2; (2) parasite positivity rates (PPRs) for the proportion of patients remaining parasitaemic on day 2; and, (3) early treatment failure (ETF) for patients with danger signs, complicated malaria or presence of parasitaemia on day 2 with fever (rectal temperature ≥38 °C) or parasitaemia on day 2 higher than on day 0. Patients were excluded from the clinical and parasitological assessment if they were lost to follow-up.

### Statistical analysis

All statistical tests were performed using GraphPad Prism software version 5 (GraphPad Inc., San Diego, CA, USA). Data that were not normally distributed were displayed as median along with interquartile ranges and were compared with Mann–Whitney U test for two group comparisons. The Gaussian data were reported as the mean ± standard deviation and were analysed using the *t* test for two group comparisons. Proportions were compared using the χ^2^ test or the Fisher’s exact test, as appropriate. Correlations were determined using the Spearman test. A comparison was considered statistically significant if the p value was ≤0.05.

## Results

### Baseline characteristics of eligible patients through the study

**Fig. 1:**
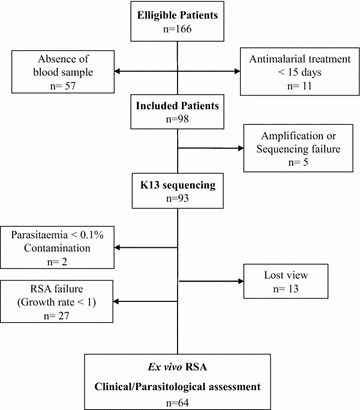
Patients’ flow through the study

Between March and June 2015, a total of 166 patients with falciparum malaria were enrolled. The flow of patients through the study is displayed in Fig. 1. The baseline characteristics of these patients are given in Table 1. The weight adjusted drug dose (mg/kg) was calculated in 98% of the patients as the body weight was available in 96 of the 98 patients. Some 76.0% of the patients (73/96) received a total dose of DHA and PPQ components, which followed WHO recommendations (DHA: 2–10 mg/kg; PPQ: 16–27 mg/kg). The 24.0% (23/96) other patients who received a dose of DHA and PPQ components under the WHO recommendations were all over 15 years old.

**Table 1:** Baseline characteristics of the patients included in the study at day 0

Baseline characteristics	
Patients (n)	98
Gender	
Sex ratio n (%)	0.96
Male	48 (49.0%)
Female	50 (51.0%)
Age	
Median age (IQR) (years)	11.5 (6–18.8)
<8 years n (%)	39 (39.8%)
8–15 years n (%)	23 (23.5%)
>15 years n (%)	36 (36.7%)
Body weight (n = 96)	
Median body weight (IQR) (kg)	33 (21–57)
<25 kg n (%)	37 (38.5%)
≥25 kg n (%)	59 (61.5%)
Temperature	
Median (IQR) (°C)	38.2 (37–39.5)
Fever (temperature ≥ 38 °C) n (%)	51 (52.0%)
Symptoms n (%)	
Headache	66 (67.3%)
Vomiting	42 (42.9%)
Diarrhoea	8 (8.2%)
Diffuse pain	9 (9.2%)
*P. falciparum* asexual parasite density	
Median parasitaemia (IQR) (parasites/µl)	42,332 (17,893–87,302)
Anti-malarial dose (mg/kg) (n = 96)	
DHA Median (IQR)	2.5 (2.0–3.0)
PPQ Median (IQR)	19.8 (16.3–24)

### *K13*-propeller polymorphisms

The *k13* gene was genotyped from 98 *P. falciparum* isolates out of the 166 eligible patients. For 57 patients, no blood sample was obtained because of blood collection difficulties or technical understaffing and 11 other patients used anti-malarial treatment within the 15 day-period before enrolment (Fig. 1). Five samples failed to amplify or did not generate high-quality sequences for analysis (Fig. 1). The *k13*-propeller sequence polymorphism analysis of the 93 isolates revealed only wild-type profiles (95% CI: 96.31–100%) with four (4.3%) synonymous changes.

### Ex-vivo ring-stage survival assay (RSA)

**Fig. 2:**
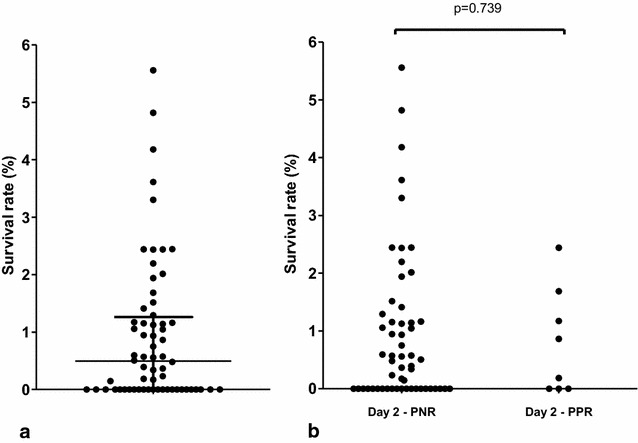
Ex-vivo survival after exposure to DHA. Ex-vivo RSA exposed 93 isolates obtained from patients with uncomplicated falciparum malaria to 700 nM DHA or dimethyl sulfoxide (DHA solvent) during 6 h. Data were considered interpretable if the growth rate was ≥1. **a** Results obtained in the ex vivo RSA from the 64 isolates with interpretable data (growth rate was ≥1) are expressed as the survival rate of *P. falciparum* parasites after a 6 hour exposure to 700 nM DHA compared with dimethyl sulfoxide (DHA solvent). The* horizontal line* represents the mean and* whiskers* the interquartile range. **b** Survival rates obtained in the ex vivo RSA from the 64 isolates with interpretable data are expressed in the two groups, day 2 PNR and day 2 PPR. Day 2 PNR represented the proportion of non-parasitaemic patients on day 2 whereas day PPR 2 represented the proportion of patients remaining parasitaemic on day 2, respectively. The ex vivo RSA data were analysed using the *t*-test for two group comparisons

DHA susceptibility data obtained for the 93 *k13*-sequenced samples were analysed using the ex vivo RSA (Fig. 1). The 64 isolates with an interpretable RSA (growth rate ≥1) had a median survival rate of 0.49% (IQR: 0–1.3) (Fig. 2). Forty of the 64 (62.5%) DHA-exposed isolates demonstrated viable parasites with a median parasitaemia of 0.012% (IQR: 0.007–0.029) and a median survival rate of 1.14% (IQR: 0.6–2.2) whereas 24 (37.5%) cultures contained only pyknotic/dead forms (Fig. 2a). The dispersion of ex vivo RSA data was high: four isolates (6.3%) had a survival rate higher than the mean survival rate plus 2 standard deviations (3.4%). There were no significant differences in the growth rate (mean = 1.878 ± 0.435 vs 2.317 ± 0.2413; *t*-test; P = 0.644) or in the initial parasitaemia (mean = 0.630 ± 0.065 vs 0.669 ± 0.052; *t*-test; P = 0.848) between parasites with a high or a low survival rate.

### Early clinical and parasitological responses to treatment

Clinical and parasitological characteristics of the studied population are shown in Table 2. The parasite clearance rate for this study was rapid as the PPR decreased to 12.5% (8/64) on day 2. The parasitaemia at day 2 in the PPR group was low with a median of 293 (IQR: 145–736) and a maximum of 1143 parasites per µl of blood. No patients presented ETF. Whereas some isolates remained parasitaemic at day 2, the rate of parasite decrease was high and varied between 97.5 and 99.9%. The PPR at day 2 was not affected by the baseline parasite density at day 0. Most patients (92.8%) were totally asymptomatic within 48 h after drug administration. No difference in age, weight, fever, DHA/PPQ anti-malarial doses or symptoms was observed between patients in day-2 PPR and PNR groups. No difference in survival rate in the ex vivo RSA was observed between the two groups, day 2 PPR and PNR (Fig. 2b).

**Table 2:** Clinical and parasitological characteristics at day 2

	Day 2 (n = 64)		P value
	PPR	PNR	
n (%)	8 (12.5%)	56 (87.5%)	
Age (years) Median (IQR)	7.5 (5–14.3)	12 (6–22)	0.236^a^
<8 years n (%)	4 (50%)	19 (33.9%)	0.893^b^
8–15 years n (%)	2 (25%)	15 (26.8%)	
>15 years n (%)	2 (25%)	22 (39.3%)	
Weight (kg) Median (IQR)	26 (18–58.3)	39 (21–61)	0.551^a^
*P. falciparum* density (parasites/µl)			
Day 0 Median (IQR)	81,260 (35,110–133,200)	44,260 (17,720–87,300)	0.150^a^
Day 2 Median (IQR)	293 (145–736)	0	
Parasite decrease rate (%) Median (IQR)	99.6 (98.9–99.8)	100	
Symptoms n (%)			
Headache	0 (0%)	3 (5.3%)	1.000^c^
Vomiting	0 (0%)	1 (1.8%)	1.000^c^
Diarrhoea	0 (0%)	0 (0%)	1.000^c^
Diffuse pain	0 (0%)	0 (0%)	1.000^c^
Fever (≥38 °C)	0 (0%)	1 (1.8%)	1.000^c^
Anti-malarial dose (mg/kg)			
DHA			
Median (IQR)	2.4 (1.9–3.1)	2.4 (2.0–2.9)	0.570^a^
<25 kg	2.5 (2.4–3.4)	2.8 (2.4–3.4)	0.891^a^
≥25 kg	2.3 (1.8–3.1)	2.1 (1.8–2.6)	0.425^a^
PPQ			
Median (IQR)	19.4 (15.5–24.6)	18.8 (15.7–22.9)	0.570^a^
<25 kg	20.0 (18.8–03.5)	22.1 (18.8–27.5)	0.891^a^
≥25 kg	18.3 (14.1–24.6)	17.1 (14.3–20.4)	0.425^a^

Day 2 PPR: proportion of patients remaining parasitaemic on day 2

Day 2 PNR: proportion of patients without parasitaemia on day 2

^a^Mann–Whitney U test

^b^Chi^2^ test

^c^Fisher’s exact test

## Discussion

The spread of resistance threatens the global control of falciparum malaria, especially if resistance reaches sub-Saharan Africa, the most malaria affected area, and this makes crucial a closely monitoring of efficacy of ACT.

Here, a genotypic (*k13* polymorphism) characterization of Cameroonian *P. falciparum* isolates was reported. Ex-vivo and in-vivo phenotypic profiles of these parasites were given for comparison in order to have a baseline picture of artemisinin susceptibility. The sequencing of the *k13*-propeller domain from samples from Cameroon did not reveal resistance genotypes and only detected synonymous mutations at a low prevalence (4.3%). The genotypic profile observed in this study was consistent with other African data that have not reported Asian profiles but only non-synonymous SNPs not associated with ART resistance [16, 19].

The ex vivo RSA performed in this study has been developed and validated in Southeast Asia to distinguish fast-clearance parasites from slow-clearance parasites in the field [15], and resistance phenotypes obtained in RSA strongly correlate with the presence of mutations in the *k13*-propeller domain [13]. The phenotypic data obtained from this study were in agreement with the genotypic ones. The absence of *k13* polymorphisms was associated with a low median of survival rate for the tested isolates (0.49%). The high dispersion of ex vivo RSA data observed in this study could not be explained by the variability in parasite clearance time as no significant difference in survival rates was observed between PPR and PNR isolates. Moreover, the four isolates with a survival rate higher than the mean plus 2 standard deviations showed no remaining parasitaemia on day 2 and had no *k13* polymorphism. These results are comparable to those observed by Ashley et al. who occasionally found patients with parasite clearance half-life values more than 5 hours but without association with *k13* polymorphisms [21]. These data were consistent with the survival rates obtained in the only ex vivo RSA study performed in East Africa (Uganda), which varied between 0.7 and 1.9% [17]. However, in their study, Cooper et al. reported only three out of 43 DHA-pulsed cultures with healthy-appearing parasites [17], which is lower than that obtained in the present work (40/64). Other factors unrelated to ART resistance could influence the dispersion in survival rate and/or a low number of positive DHA-pulsed cultures, such as a lower growth rate during the ex vivo RSA or a difference in parasite age or density at the initiation of the test. Indeed Witkowski et al. pointed out in Cambodia that a growth rate higher than 1 is required to correctly analyse survival rate from ex vivo RSA [15]. Witkowski et al. also showed that RSA performed with late-ring or trophozoite forms does no longer identify a slow-clearance infection, by contrast to RSA with early-ring forms [15]. So storage and transport conditions of parasite isolates before processing are highly important in order to have the earliest parasite stages and then to obtain the more reliable survival rates in the ex vivo RSA.

In this study, the genotypic and ex vivo phenotypic profiles were associated with a rapid parasite reduction ratio. At day 2, 87.5% (56/64) of patients had no detectable parasitaemia, the remaining 12.5% had a low *P. falciparum* density and the prevalence of clinical symptoms was highly reduced for all patients. Simple measure of parasitaemia is a good approximation of the parasite clearance rate [21]. In this study, the clinical and parasitological assessment was chosen to be based on a follow-up with two time points, at enrolment (day 0) and at day 2, regarding the fast parasite clearance time observed in Africa [23–25] as in the Nkol-Eton healthcare centre. Despite a possible correlation between parasitaemia at day 2/3 and at enrolment [26], the assessment of the proportion of patients with a detectable parasitaemia at day 2 seemed a relevant parameter for quantitatively comparing with genotypic and ex vivo phenotypic profiles, and for rapidly detecting any change in *P. falciparum* susceptibility. Moreover this follow-up is easier to implement in healthcare centres compared to the six measurements within the first 48 h of treatment that require hospitalization of patients and a dedicated staff.

This study finally shows the feasibility of an overall, effective and inexpensive assessment of the susceptibility of *P. falciparum* to artemisinin derivatives that could be implemented in most endemic countries with minimal equipment (listed in “Methods” section, with the exception of sequencing that can be achieved at a later stage) but with experienced and well-trained technicians for the correct execution of the RSA.

## Conclusions

This study gives reassurance about the level of ART resistance in Cameroon and provides baseline data of *k13* polymorphism, survival rate in ex vivo RSA and PPR profiles that will be essential for monitoring ART-derivative susceptibility in Africa. Similar studies will be required in other African countries to obtain an exhaustive view of these phenotypic data.

## Abbreviations

ACD: acid-citrate-dextrose
ACT: artemisinin-based combination therapy
ART: artemisinin
DHA: dihydroartemisinin
DNA: deoxyribonucleic acid
DMSO: dimethyl sulfoxide
ETF: early treatment failure
IC50: 50% inhibitory concentration
IC95: 95% confidence interval
IQR: interquartile range
PCR: polymerase reaction chain
PNR: parasite negative rate
PPR: parasite positive rate
PPQ: piperaquine
RSA: ring-stage survival assay
SNP: single nucleotide polymorphism
WHO: World Health Organization
